# Single nucleotide polymorphism rs7961894, platelet morphological parameters and lipid profile in children with type 1 diabetes: a potential relationship

**DOI:** 10.1007/s00431-024-05694-1

**Published:** 2024-08-05

**Authors:** Mahmoud A. El-Hawy, Shimaa Abdelsattar, Hanan M. Bedair, Doaa Z. Elsaady, Ahmed S. Abo Hola

**Affiliations:** 1 Department of Pediatrics, Faculty of Medicine, Menoufia University, Yassin Abdel-Ghafar Street, Shebin El-Kom, Egypt; 2 Clinical Biochemistry and Molecular Diagnostics Department, National Liver Institute, Menoufia University, Shebin El-Kom, Egypt; 3 Clinical Pathology Department, National Liver Institute, Menoufia University, Shebin El-Kom, Egypt; 4 Egyptian Ministry of Health, Cairo, Egypt

**Keywords:** Lipid profile, Mean platelet volume, Platelet activity, Single nucleotide polymorphism, rs7961894, Type 1 diabetes

## Abstract

Increased cardiovascular risk has been associated with certain platelet morphological parameters, and several single nucleotide polymorphisms (SNPs) have been reported to be linked. Still, little is known about their role among children with type 1 diabetes mellitus (T1DM). So, we aimed to investigate platelet parameters and lipid profile changes in relation to rs7961894 SNP in children with T1DM. Eighty children with T1DM and eighty apparently healthy controls participated in this cross-sectional study. Platelet count, mean platelet volume (MPV), platelet distribution width (PDW), plateletcrit (PCT), HbA1c, triglycerides, total cholesterol (TC), low-density lipoprotein cholesterol (LDL-C), and high-density lipoprotein cholesterol were measured, and atherogenic indices were calculated. Using a real-time polymerase chain allelic discrimination technique, rs7961894 SNP was genotyped. Children with T1DM had significantly higher MPV, PDW, TC, and LDL-C compared to controls. 25% of patients had rs7961894 CT genotype with significantly higher MPV, PDW, PCT, LDL-C, triglycerides, Castelli’s risk index II (CRI II), and atherogenic index of plasma (AIP) compared to CC genotyped patients. MPV correlated significantly with CRI II and AIP, PDW with CRI II, while PCT correlated substantially with HbA1c, LDL-C, CRI II, and AIP. rs7961894 CT genotype was a significant dependent predictor of the changes in MPV, PDW, and PCT in multivariate regression analysis.

*Conclusion*: In children with T1DM, rs7961894 CT genotype is significantly linked to MPV, PDW, and PCT changes, which showed a substantial relationship to CRI II and AIP, highlighting the importance of monitoring these patients to identify potential cardiovascular risks early.

**Table:** 

**What is Known:** • *Platelets and dyslipidemia are involved in atherosclerosis pathogenesis* • *Changes in platelet activity and morphological parameters in diabetes mellitus are contradictory* • *rs7961894 single nucleotide polymorphism is associated with significant changes in mean platelet volume (MPV) with no available data in children*
**What is New:** • *Children with type 1 diabetes mellitus exhibited significantly higher values of MPV and platelet distribution width (PDW)* • *rs7961894 CT genotype was a dependent predictor of the changes in MPV, PDW, and plateletcrit (PCT) values* • *Diabetic children with the rs7961894 CT genotype showed substantial alterations in lipid parameters with a strong correlation between MPV, PDW, and PCT and Castelli’s risk index II and the atherogenic index of plasma*

**What is Known:**

• *Platelets and dyslipidemia are involved in atherosclerosis pathogenesis*

• *Changes in platelet activity and morphological parameters in diabetes mellitus are contradictory*

• *rs7961894 single nucleotide polymorphism is associated with significant changes in mean platelet volume (MPV) with no available data in children*

**What is New:**

• *Children with type 1 diabetes mellitus exhibited significantly higher values of MPV and platelet distribution width (PDW)*

• *rs7961894 CT genotype was a dependent predictor of the changes in MPV, PDW, and plateletcrit (PCT) values*

• *Diabetic children with the rs7961894 CT genotype showed substantial alterations in lipid parameters with a strong correlation between MPV, PDW, and PCT and Castelli’s risk index II and the atherogenic index of plasma*

## Introduction

Type 1 diabetes mellitus (T1DM) is one of the most common endocrinological disorders in children and adolescents. Its annual incidence in children under 14 ranges from 0.1/100,000 to 36.8/100,000, depending on the country [1]. It has been shown that platelets in diabetic patients are more adhesive and aggregative, both independently and in response to stimulants [2]. Since platelet activation is a primary cause of atherosclerosis, children with T1DM may experience microvascular and macrovascular consequences. Meanwhile, other factors like diabetes duration, glycemic control efficacy, dyslipidemia, obesity, stress, and genetic predisposition may also be at play [3].

Platelet volume and functions can be represented by mean platelet volume (MPV) and platelet distribution width (PDW), and despite PDW being more specific, MPV is regarded as a marker linked to increased platelet activity. Plateletcrit (PCT), a measure of total platelet mass, has also been found to be higher in patients with thrombosis [4].

Hyperglycemia is hypothesized to directly affect platelet membrane glycation, which could be a factor in the increased platelet activity. Nevertheless, it is still unclear how metabolic control affects platelet morphologic characteristics, particularly in T1DM patients, and inconsistent results have been found in multiple investigations [5, 6].

There is compelling evidence that dyslipidemia induces platelet activation based on “in vitro” mechanistic investigations on platelet reactivity and severe adverse cardiac events in patients with coronary artery disease (CAD) [7]. Serum lipid abnormalities have been reported in children with T1DM even with excellent glycemic control, and variations in ethnicity, diet, lifestyle, pubertal influences, or even genetic factors could account for this [8, 9].

Significant diversity in platelet functions between different individuals is influenced by both genetic and environmental variables, and several single nucleotide polymorphisms (SNPs) have been linked to platelet indices, especially MPV, by genome-wide association studies (GWAS) [10, 11]. While there is currently no data in pediatrics, the rs7961894 SNP is associated with significant MPV changes, and a higher risk of stroke in adults [12].

Therefore, in this study, our goal was to evaluate platelet parameters and lipid profile alterations in relation to rs7961894 SNP in children with T1DM.

## Subjects and methods

### Subjects

Eighty children diagnosed with T1DM from Pediatric Endocrinology Clinic at Menoufia University Hospitals were enrolled in this study, with eighty apparently healthy age and sex matched children as controls. Patients with hypoglycemia, diabetic ketoacidosis, acute or chronic infections, inherited or acquired diseases that may affect platelet counts or functions, associated other chronic diseases, and concomitant drug use such as lipid-lowering or non-steroid anti-inflammatory agents which may interfere with the study were excluded.

### Methods

After the consent letter was approved by the patients’ caregivers, all patients were subjected to a detailed history and a thorough clinical examination to exclude any associated criteria that may interfere with the study.

Under aseptic conditions, fasting venous blood samples were withdrawn from all participants in ethylenediaminetetraacetic acid (EDTA) tubes and processed at room temperature within 2 h. Complete blood counts were analyzed through Sysmex XT-1800i Automated Hematology Analyzer, and platelet parameters (platelet count, MPV, PDW, and PCT) were recorded. HbA1c assay was performed through cobas c 311 auto analyzer, Roche diagnostics, Germany. Lipid profile parameters including [total cholesterol (TC), triglycerides (TG), low-density lipoprotein cholesterol (LDL-C), and high-density lipoprotein cholesterol (HDL-C)] were measured by using AU 680 Beckmann autoanalyzer, and subsequently, atherogenic indices [non-HDL-C; TC-HDL-C, Castelli’s risk index I (CRI I); TC/HDL-C, Castelli’s risk index II (CRI II); LDL-C/HDL-C, atherogenic index of plasma (AIP); Log TG/ HDL-C, and atherogenic coefficient (AC); non-HDL-C/ HDL-C] were calculated [13].

Furthermore, 1 ml of whole blood was added to EDTA tubes and stored in -20 ºC for subsequent genomic DNA extraction, which was performed using Gene JET TM whole blood Genomic DNA purification Mini kit [Thermo Scientific EU/Lithuania] according to the manufacturer’s instructions. Genotyping of the rs7961894 SNP was performed by real-time polymerase chain allelic discrimination technology using TaqMan SNP genotyping assay kit [Thermo Fisher Scientific, Waltham, MA, United States]. Allelic discrimination was manifested by TaqMan fluorogenic minor groove binder probes. Polymerase chain reaction data analysis was conducted on Applied Biosystem 7500 RT-PCR ABI PRISM (Applied Biosystems, United States) software v.2.0.1. For the examined polymorphism, the genotype distribution complied with the Hardy–Weinberg equilibrium.

### Statistical analysis

Utilizing IBM SPSS Statistics Version 20.0. (IBM Corp, Armonk, NY), results were statistically analyzed. Descriptive data were presented using the mean ± standard deviation, median, interquartile range (IQR), and percent. Mann–Whitney U test and t test were used to compare the means of non-normally and normally distributed quantitative data, respectively, and Chi-square test analyzed qualitative variables. Spearman’s coefficient was used to evaluate the associations between studied parameters. Regression analysis was used to assess variables that may affect platelet parameters. Statistics were deemed significant with a p-value ≤ 0.05.

With 0.8 statistical power and a 95% confidence level, and according to a previous study [14], the sample size was determined to be a minimum of 67 participants using power analysis techniques to identify group differences larger than 0.2 standard deviation.

## Results

Children with T1DM reported a mean age of 13.39 ± 2.81 years, with a non-statistical difference to that of controls (13.43 ± 2.97 years). They exhibited a statistically lower weight and height, and despite being within the normal range, their systolic and diastolic blood measurements were significantly higher than controls **(**Table 1**)**.

**Table 1:** Comparison of clinical and laboratory data between patients and controls

Variables	Patients (*n* = 80)		Control (*n* = 80)		*P* value
Sex	No	%	No	%	0.079
Male	29	36.3	40	50.0	
Female	51	63.8	40	50.0	
Age (years) Mean ± SD	13.39 ± 2.81		13.43 ± 2.97		0.943
Weight Z–Score Mean ± SD	-0.45 ± 0.74		-0.17 ± 0.54		0.009*
Height z score Mean ± SD	-0.68 ± 0.71		-0.43 ± 0.49		0.041*
BMI z score Mean ± SD	-0.32 ± 1.02		-0.02 ± 0.74		0.128
Systolic blood pressure (mmHg) Mean ± SD	120.3 ± 5.57		110.0 ± 5.88		< 0.001**
Diastolic blood pressure (mmHg) Mean ± SD	76.80 ± 3.08		63.25 ± 2.38		< 0.001**
Hemoglobin (g/dl) Mean ± SD	13.71 ± 1.41		13.41 ± 1.28		0.154
Red blood cell count (10^6^/mm^3^) Mean ± SD	5.24 ± 0.63		5.12 ± 0.70		0.271
White blood cell count (10^3^/mm^3^) Mean ± SD	9.05 ± 2.28		8.92 ± 2.15		0.530
Platelet count (10^3^/mm^3^) Mean ± SD	309.6 ± 69.26		313.4 ± 73.21		0.844
Plateletcrit % Mean ± SD	0.32 ± 0.12		0.30 ± 0.13		0.083
Mean platelet volume (fL) Mean ± SD	9.87 ± 2.30		8.15 ± 0.88		< 0.001**
Platelet distribution width % Mean ± SD	15.91 ± 4.02		9.89 ± 1.73		< 0.001**
HbA1c % Mean ± SD	11.07 ± 1.89		4.85 ± 0.32		< 0.001**
Total cholesterol (TC) mg/dl Mean ± SD	127.9 ± 4.53		121.7 ± 17.67		0.003*
High-density lipoprotein cholesterol (LDL-C) mg/dl Mean ± SD	61.64 ± 2.52		60.69 ± 4.54		0.104
Low-density lipoprotein cholesterol (HDL-C) mg/dl Mean ± SD	88.80 ± 4.37		85.25 ± 8.55		0.001*
Triglycerides (TG) mg/dl Mean ± SD	79.75 ± 13.31		78.09 ± 7.98		0.340
non-HDL-C (mg/dl) Mean ± SD	66.23 ± 5.13		61.00 ± 18.06		0.015*
Castell’s risk index I (TC/HDL-C) Mean ± SD	2.08 ± 0.11		2.01 ± 0.32		0.095
Castell’s risk index II (LDL-C/HDL-C) Mean ± SD	1.44 ± 0.10		1.41 ± 0.17		0.130
Atherogenic index of plasma (log TG/HDL-C) Mean ± SD	0.11 ± 0.08		0.11 ± 0.05		0.806
Atherogenic coefficient (non-HDL-C/HDL-C) Mean ± SD	1.08 ± 0.11		1.01 ± 0.32		0.095

SD: standard deviation, *: Statistically significant at p ≤ 0.05, **: highly Statistically significant at *p* value < 0.001

Regarding hematological parameters, patients had significantly higher MPV and PDW compared to controls, with no significant differences detected in hemoglobin levels, red blood cell counts (RBCs), white blood cell counts (WBCs), platelet counts, and PCT between the studied groups. HbA1c, TC, LDL-C, and non-HDL-C were statistically higher in patients, with no significant differences regarding other lipid parameters **(**Table 1).

Genotyping of rs7961894 documented a CT genotype frequency of 25% in patients compared to 12.5% in controls, with a significant statistical difference. In addition, odd ratio analysis determined that individuals with the CT genotype were 2.333 times more risky to have T1DM than those with the wild CC genotype **(**Table 2**)**.

**Table 2:** rs7961894 genotypes and allele distribution among patients and controls

	Patients		Controls		p value	OR (LL – UL 95%C.I)
	***No***	**%**	***No***	**%**		
rs7961894 genotypes	**(*n*** **= 80)**		**(*n*** **= 80)**			
CC	60	75.0	70	87.5		1.000
CT	20	25.0	10	12.5	0.046*	2.333(1.014 – 5.371)
TT	0	0.0	0	0.0	–	–
rs7961894 allele distribution	**(*n*** **= 160)**		**(*n*** **= 160)**			
C	140	87.50	150	93.8		1.000
T	20	12.5	10	6.3	0.060	2.143(0.969 – 4.737)

C.I: Confidence interval, LL: Lower limit, UL: Upper Limit, OR: odd ratio, *: Statistically significant at *p* ≤ 0.05

Patients with the CT genotype had a statistically higher MPV, PDW, PCT, HbA1c, LDL-C, TG, CRI II, and AIP compared to those with the CC genotype, with no significant differences detected between them regarding other parameters (Table 3).

**Table 3:** Comparison of clinical and laboratory data in patients according to rs7961894 genotyping

	CC genotyped patients (*n* = 60)		CT genotyped patients (*n* = 20)		*p* value
Sex	**No**	**%**	**No**	**%**	
Male	23	38.3	6	30.0	0.502
Female	37	61.7	14	70.0	
Age (years) Mean ± SD	13.53 ± 2.74		12.95 ± 3.05		0.438
Family history					
Negative	54	90.0	20	100.0	0.328
Positive	6	10.0	0	0.0	
Age of onset of DM Mean ± SD	6.68 ± 1.90		5.95 ± 1.96		0.097
Duration of onset of DM Mean ± SD	6.83 ± 1.65		7.0 ± 2.41		0.829
Weight Z–Score Mean ± SD	-0.50 ± 0.78		-0.30 ± 0.63		0.333
Height Z–Score Mean ± SD	-0.74 ± 0.70		-0.50 ± 0.73		0.053
BMI Z–Score Mean ± SD	-0.35 ± 1.06		-0.26 ± 0.94		0.982
Systolic blood pressure (mmHg) Mean ± SD	120.8 ± 5.53		118.8 ± 5.57		0.177
Diastolic blood pressure (mmHg) Mean ± SD	76.93 ± 3.04		76.40 ± 3.23		0.506
Hemoglobin (g/dl) Mean ± SD	13.68 ± 1.44		13.81 ± 1.33		0.730
Red blood cell count (10^6^/mm^3^) Mean ± SD	5.26 ± 0.58		5.17 ± 0.78		0.580
White blood cell count (10^3^/mm^3^) Mean ± SD	9.07 ± 2.28		8.99 ± 2.34		0.938
Platelet count (10^3^/mm^3^) Mean ± SD	302.4 ± 64.37		331.5 ± 80.04		0.276
Plateletcrit % Mean ± SD	8.93 ± 1.65		12.72 ± 1.48		< 0.001**
Mean platelet volume (fL) Mean ± SD	14.40 ± 3.18		20.43 ± 2.68		< 0.001**
Platelet distribution width % Mean ± SD	0.28 ± 0.10		0.43 ± 0.11		< 0.001**
HbA1c % Mean ± SD	10.41 ± 1.51		13.06 ± 1.48		< 0.001**
Total cholesterol (TC) mg/dl Mean ± SD	127.8 ± 4.96		128.1 ± 2.98		0.720
High-density lipoprotein cholesterol (LDL-C) mg/dl Mean ± SD	61.74 ± 2.40		61.34 ± 2.89		0.543
Low-density lipoprotein cholesterol (HDL-C) mg/dl Mean ± SD	86.99 ± 2.52		94.22 ± 4.27		< 0.001**
Triglycerides (TG) mg/dl Mean ± SD	77.22 ± 12.82		87.35 ± 12.04		0.003*
non-HDL-C (mg/dl) Mean ± SD	66.04 ± 5.55		66.77 ± 3.66		0.584
Castell’s risk index I (TC/HDL-C) Mean ± SD	2.07 ± 0.12		2.09 ± 0.10		0.497
Castell’s risk index II (LDL-C/HDL-C) Mean ± SD	1.41 ± 0.07		1.54 ± 0.10		< 0.001**
Atherogenic index of plasma (log TG/HDL-C) Mean ± SD	0.09 ± 0.08		0.15 ± 0.06		0.001*
Atherogenic coefficient (non-HDL-C/HDL-C) Mean ± SD	1.07 ± 0.12		1.09 ± 0.10		0.497

SD: standard deviation, *: Statistically significant at *p* ≤ 0.05, **: highly Statistically significant at *p* value < 0.001

**Fig. 1:**
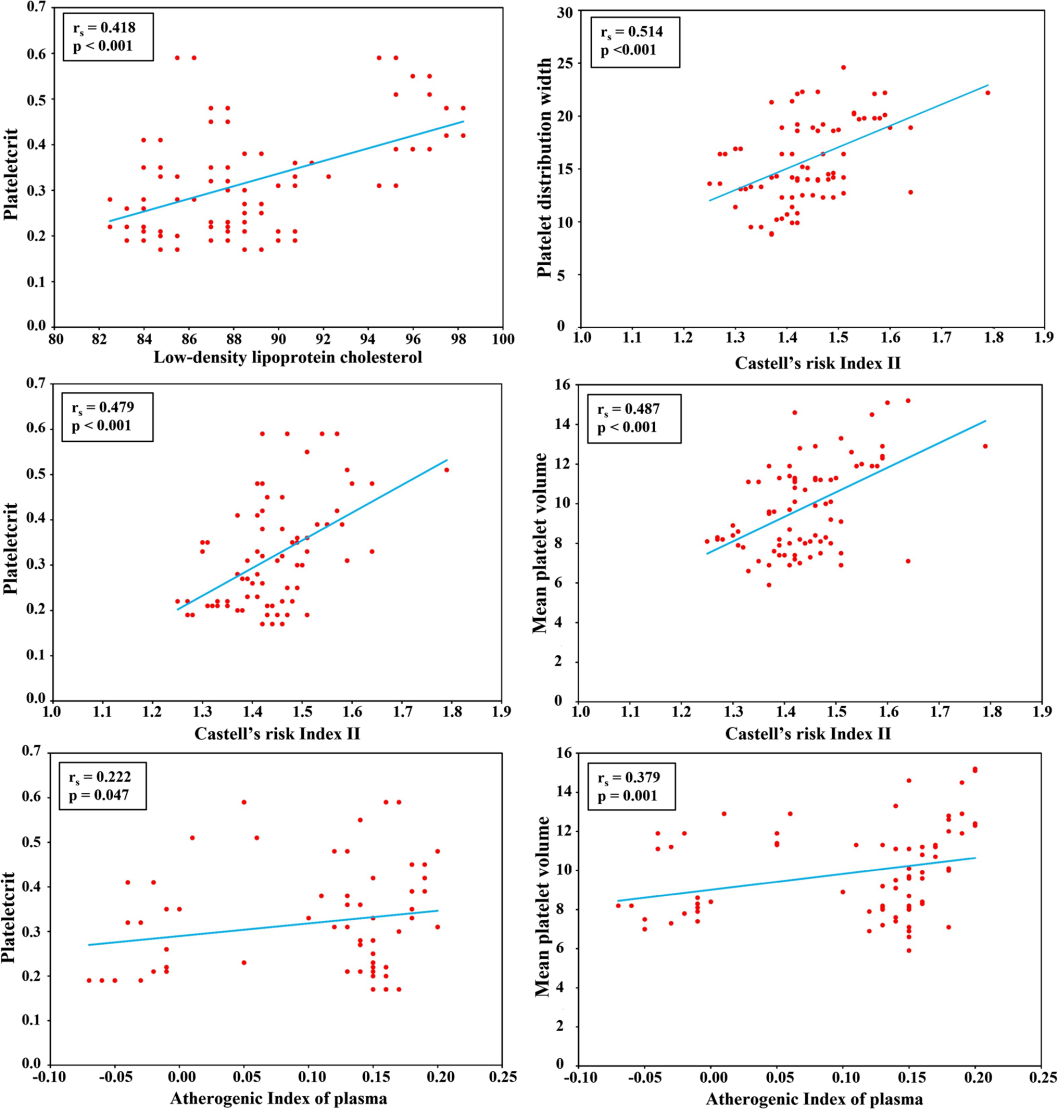
Correlations between platelet morphological parameters and lipid parameters

Platelet count was significantly correlated with Hb level, RBCs, WBCs, and PCT. Also, PCT was significantly correlated with HbA1c and LDL-C. MPV, PDW, and PCT correlated well with each other, and with CRI II as well. AIP correlated significantly only to MPV and PCT (Table 4, Fig. 1).

**Table 4:** Correlations between platelet parameters and other laboratory data in patients

Variables	Platelet count (10^9^/L)		Plateletcrit %		Mean platelet volume (fL)		Platelet distribution width %	
	**r**_**s**_	**p value**	**r**_**s**_	**p value**	**r**_**s**_	**p value**	**r**_**s**_	**p value**
Platelet count (10^3^/mm^3^)	–	–						
Plateletcrit %	0.614	< 0.001**	–	–				
Mean platelet volume (fL)	0.130	0.249	0.547	< 0.001**	–	–		
Platelet distribution width %	0.088	0.440	0.564	< 0.001**	0.845	< 0.001**	–	–
Hemoglobin (g/dl)	0.251	0.024*	0.353	0.001*	-0.034	0.762	0.119	0.292
Red blood cell count (10^6^/mm^3^)	0.277	0.013*	0.032	0.776	-0.071	0.530	-0.119	0.293
White blood cell count (10^3^/mm^3^)	0.269	0.016*	0.361	0.001*	0.214	0.057	0.163	0.148
HbA1c %	0.080	0.482	0.263*	0.018*	0.460	< 0.001*	0.286*	0.010*
Total cholesterol (TC) mg/dl	-0.170	0.131	-0.048	0.674	-0.056	0.619	0.125	0.270
High-density lipoprotein cholesterol (HDL-C) mg/dl	-0.093	0.414	-0.171	0.129	-0.118	0.299	-0.082	0.469
Low-density lipoprotein cholesterol (LDL-C) mg/dl	-0.009	0.936	0.418	< 0.001**	0.446	0.000	0.504	0.000
Triglycerides (TG) mg/dl	-0.061	0.589	0.197	0.080	0.401	0.000	0.185	0.100
non-HDL-C (mg/dl)	-0.076	0.504	0.050	0.658	0.024	0.834	0.154	0.171
Castell’s risk index I (TC/HDL-C)	-0.010	0.928	0.102	0.368	0.040	0.724	0.144	0.204
Castell’s risk index II (LDL-C/HDL-C)	0.059	0.600	0.479	< 0.001**	0.487	< 0.001**	0.514	< 0.001**
Atherogenic index of plasma (log TG/HDL-C)	-0.084	0.456	0.222	0.047*	0.379	0.001*	0.170	0.131
Atherogenic coefficient (non-HDL-C/HDL-C)	-0.010	0.928	0.102	0.368	0.040	0.724	0.144	0.204

* Statistically significant at *p* ≤ 0.05, ** highly Statistically significant at *p* value < 0.001

In multivariate regression analysis, WBCs and the rs7961894 CT genotype were dependent predictors of MPV, PDW, and PCT changes, while Hb was a dependent marker for platelet count and PCT changes (Table 5).

**Table 5:** Univariate and multivariate regression analysis for variables affecting changes in platelet parameters in patients

	Univariate		Multivariate	
	*p* value	B (LL – UL 95%C.I)	*p* value	B(LL – UL 95%C.I)
Variables for platelet count				
Hemoglobin	0.005*	14.989(4.428 – 25.549)	0.003*	16.905(0.739 – 1.353)
Red blood cell count	0.020*	28.369(4.595 – 52.143)	0.412	9.813(0.797 – 1.254)
White blood cell count	0.023*	7.730(1.111 – 14.349)	0.254	3.780(0.805 – 1.242)
Variables for mean platelet volume				
White blood cell count	0.045*	0.227(0.006 – 0.449)	0.002*	0.237(0.087 – 0.388)
HbA1c	< 0.001**	0.594(0.354 – 0.835)	0.637	0.059(-0.190 – 0.308)
Low-density lipoprotein cholesterol (LDL-C)	< 0.001**	0.320(0.226 – 0.415)	0.334	0.074(-0.078 – 0.226)
Triglycerides (TG)	0.008*	0.051(0.013 – 0.088)	0.815	0.003(-0.026 – 0.033)
Castell’s risk Index II (LDL-C/HDL-C)	< 0.001**	12.443(7.865 – 17.021)	0.490	1.996(-3.738 – 7.729)
rs7961894 CT genotype	< 0.001*	3.788(2.960 – 4.617)	< 0.001**	2.826(1.505 – 4.147)
Variables for platelet distribution width				
White blood cell count	0.045*	0.227(0.006 – 0.449)	0.002*	0.240(0.090 – 0.390)
HbA1c	0.007*	0.633(0.174 – 1.092)	0.662	0.054(-0.192 – 0.300)
Low-density lipoprotein cholesterol (LDL-C)	< 0.001**	0.320(0.226 – 0.415)	0.067	0.107(-0.008 – 0.222)
Atherogenic index of plasma (log TG/HDL-C)	0.009*	8.156(2.075 – 14.237)	0.749	0.766(-3.979 – 5.511)
rs7961894 CT genotype	< 0.001**	6.027(4.452 – 7.601)	< 0.001**	2.844(1.528 – 4.160)
Variables for plateletcrit				
Hemoglobin	0.001*	0.029(0.012 – 0.047)	0.014*	0.018(0.004 – 0.032)
White blood cell count	< 0.001**	0.020(0.009 – 0.031)	< 0.001**	0.018(0.010 – 0.027)
HbA1c	0.014*	0.017(0.004 – 0.031)	0.423	-0.005(-0.018 – 0.008)
Total cholesterol (TC)	< 0.001**	0.005(0.003 – 0.008)	0.764	-0.001(-0.005 – 0.003)
High-density lipoprotein cholesterol (HDL-C)	< 0.001**	-0.022(-0.029 – -0.015)	0.549	-0.002(-0.010 – 0.006)
Low-density lipoprotein cholesterol (LDL-C)	< 0.001**	0.010(0.006 – 0.014)	0.052	0.007(0.0 – 0.013)
rs7961894 CT genotype	< 0.001**	0.145(0.094 – 0.197)	0.011*	0.102(0.025 – 0.180)

B: Unstandardized Coefficients, C.I: Confidence interval, LL: Lower limit, UL: Upper Limit, * Statistically significant at *p* ≤ 0.05, ** highly Statistically significant at *p* value < 0.001

## Discussion

T1DM is a chronic condition with rising prevalence and variable incidence rates among societies, and despite newly developed therapeutic options, chronic T1DM complications are becoming more common with the rising atherosclerotic risk [15, 16]. It has been demonstrated that diabetes is a prothrombotic state with increased platelet activation due to altered platelet membrane glycation and an associated increase in proinflammatory markers [17]. Furthermore, recent research has shown that platelet indices directly impact platelet activity and represent an emerging risk factor for vascular thrombotic complications, which are poorly studied in T1DM [1, 18].

Studies evaluating platelet counts in diabetic individuals have yielded inconsistent findings, possibly due to variations in platelet production and turnover. Raised platelet counts could be a result of stress or reactive thrombocytosis, which may be linked to poor glycemic control [19, 20]. Reduced platelet counts may be the consequence of either ineffective thrombopoiesis or an overreaction of platelets to endogenous agonists, depleting them in the circulation [21]. Others found no significant changes in platelet counts in diabetic individuals [1, 18, 22, 23]. Similarly, our findings documented no discernible difference between children with T1DM and controls regarding platelet counts.

Larger platelets adhere more easily, have denser granules, and produce more thrombotic agents than smaller ones, which is proved by “in vitro” aggregation. The parameters that reflect platelet volume are MPV and PDW [4, 23]. MPV is more frequently used to evaluate platelet size, and several studies reported significantly higher MPV values in children with T1DM than in healthy controls, whether or not this difference was related to glycemic control [3, 21, 24]. Even Brown et al. pointed out that elevated MPV in individuals with T1DM may be more suggestive of atherosclerosis than of diabetes [25]. Meanwhile, other research found no connection between diabetes and MPV [1, 17, 26]. Regarding PDW, Sharma et al. observed significantly elevated MPV and PDW values in patients with T2DM with a significant correlation to diabetes duration and glycemic control status [27]. In T1DM, Malachowska et al. determined an increase in PDW values in patients compared to a control group [28], but others disagreed [1, 29]. In this instance, our study showed that children with T1DM had a statistically higher MPV and PDW when compared to healthy controls.

PCT is assumed to represent platelet heterogeneity and to display the number of platelets in circulation in a single unit volume. Few studies have examined PCT values in diabetes; Korkmaz and Venkatesh et al. observed no discernible variation in PCT values between children with T1DM and controls. However, higher PCT values were seen in another investigation with T2DM patients, especially when associated with chronic complications [30]. Regarding our findings, no significant difference in PCT values was detected between patients and controls.

The non-enzymatic glycosylation of proteins on the platelet surface caused by hyperglycemia has a negative impact on membrane fluidity and raises platelet reactivity as a result. A little research has looked at the relationships between platelet morphological parameters and metabolic regulation in children with type 1 diabetes, still the reported data is contentious, whether it is supportive or not [31–33]. In this context, our results detected no significant correlations between HbA1c levels and platelet parameters except for PCT, which was consistent with Korkmaz’s findings [1].

This controversy could possibly be clarified by considering that factors other than a metabolic imbalance may also contribute to variations in platelet parameters in children with diabetes. Peripheral platelets have been reported to be activated in patients with a new diabetes diagnosis or are even pre-diabetic [28], which may be attributed to a possible genetic basis or an increase in proinflammatory markers that may impact progenitor cells.

Genes play a major role in determining MPV, and several SNPs have been linked to MPV by GWAS [10, 11]. In CAD, a higher MPV has been linked to greater short- and long-term mortality, and there is growing interest in using MPV-associated SNPs as CAD prognostic markers [4, 34]. Meisinger et al. observed a noteworthy correlation between MPV and the transcript level of the WDR 66 gene, which is implicated in the production of platelets [35]. In this instance, various GWAS conducted in the European population have indicated that rs7961894 SNP, which is located in intron 3 of the WDR66 gene, has a powerful association with MPV [36–38]. Furthermore, Miller et al. reported that MPV is a measure of the severity of a stroke, and the T > C variant of rs7961894 is linked to lower 1-year mortality after a stroke and is independently associated with higher MPV during the acute phase of an ischemic stroke [12].

In this context, our study showed that 25% of children with T1DM had a CT genotype for rs7961894 with a significant statistical difference when compared to healthy controls. Also, the odd ratio documented a significantly 2.333 times higher risk for T1DM in patients carrying rs7961894 CT genotyping. Those patients had a significantly higher MPV, PDW, and PCT when compared to those carrying CC genotype. Furthermore, rs7961894 CT genotype was a dependent predictor of changes in MPV, PDW, and PCT in multivariate regression analysis. This emphasizes how crucial it is to determine the genetic components associated with platelet characteristics because variations in these characteristics have been associated with an increased risk of microvascular and macrovascular complications.

Dyslipidemia is a major risk factor in the development of atherosclerosis through chronic accumulation of lipid-rich plaque in vascular walls, and platelets have been demonstrated to play a part in this through the initiation and propagation of atherosclerotic plaques [39, 40]. A substantial relationship between MPV and TC, TG, LDL-C, and HDL-C was found in an adult type 2 diabetes study [41]. In T1DM patients, subtle lipid abnormalities may be encountered despite adequate glycemic control, which may hasten the development of vascular lesions [8, 9]. Despite this, data on lipid profile alterations and their connection to platelet parameters in children with T1DM is very lacking.

In our study, children with T1DM had significantly elevated TC, LDL-C, and non-HDL-C levels when compared to healthy controls. Moreover, patients with the rs7961894 CT genotype had significantly elevated LDL-C and TG when compared to those with the CC genotype. Additionally, only PCT correlated significantly with LDL-C levels.

Some studies suggested that calculated lipid ratios are more closely related to atherosclerosis progression than any other single lipid parameter [42, 43]. In angiographically CAD verified cases, Bhardwaj et al. found that AIP, CRI I, CRI II, and AC were highly elevated and greatly increased the risk of CAD, with AIP contributing 31%, CRI I contributing 20%, AC contributing 17%, and CRI II contributing 20% [44]. Additionally, AIP was found to be substantially higher in T2D patients and to be connected with cardiovascular risk factors, according to Lumu et al. [45].

In this concern, we observed that diabetic children with the rs7961894 CT genotype had significantly elevated CRI II and AIP when compared to those with the CC genotype. Furthermore, MPV and PCT correlated significantly with CRI II and AIP, while PDW correlated significantly only with CRI II. These results suggest the necessity for additional extensive research regarding the involvement of the rs7961894 SNP in lipid profile alterations and their connection to platelet morphological parameters.

## Study limitations and strengths

Our study’s primary limitation was the small sample size, particularly with no funding support. Moreover, there is a dearth of data in this field to compare with. Nevertheless, to the best of our knowledge, this study is the first to analyze the rs7961894 SNP in children with T1DM and correlate it with changes in lipid profiles and platelet parameters in these patients.

## Conclusion

The current study highlights that the rs7961894 SNP CT genotype is linked to higher values of MPV, PDW, PCT, LDL-C, TG, CRI II, and AIP. These parameters are all strongly correlated with an increased risk of atherosclerosis according to different studies. Therefore, they should be closely monitored for the early identification of any potential microvascular and macrovascular consequences in children with T1DM as atherosclerotic diseases are conditions that can be prevented.

## Data Availability

The datasets used and/or analyzed during the current study are available from the corresponding author on reasonable request.

## Abbreviations

AIP: Atherogenic index of plasma
CRI II: Castelli’s risk index II
LDL-C: Low-density lipoprotein cholesterol
MPV: Mean platelet volume
PDW: Platelet distribution width
PCT: Plateletcrit
SNPs: Single nucleotide polymorphisms
T1DM: Type 1 diabetes mellitus
TC: Total cholesterol
